# Isoliquiritigenin in Breast Cancer: A Systematic Review of Its Preventive and Anti-metastatic Mechanisms

**DOI:** 10.5812/ijpr-165301

**Published:** 2025-10-12

**Authors:** Yuan Xue, Mingjun Wang

**Affiliations:** 1 Department of Thyroid and Breast Surgery, The First Affiliated Hospital of Henan, University of Science and Technology, Luoyang, China

**Keywords:** Isoliquiritigenin, Breast Cancer, Metastasis, Apoptosis, Nanoparticle Delivery

## Abstract

**Context:**

Breast cancer is the most prevalent malignancy among women globally, with metastasis significantly reducing survival rates. Isoliquiritigenin (ISL), a bioactive chalcone derived from *Glycyrrhiza* species, has shown promise in preclinical studies for its multifaceted anticancer properties, including modulation of metastatic processes.

**Objectives:**

This systematic review evaluates preclinical evidence on ISL’s mechanisms in breast cancer prevention and metastasis suppression.

**Evidence Acquisition:**

Following PRISMA guidelines, a comprehensive search was conducted across PubMed/Medline, Scopus, Embase, and grey literature up to May 2025. Quality was assessed using Grading of Recommendations Assessment, Development, and Evaluation (GRADE) for in vitro studies and SYRCLE’s Risk of Bias (RoB)/Animal Research: Reporting of in vivo Experiments (ARRIVE) for in vivo studies.

**Results:**

From 4,522 records, 33 studies (52 datasets: One in situ, 33 in vitro, 18 in vivo) met inclusion criteria. Most studies originated from China and Hong Kong, with robust methodological quality, though reporting on randomization and blinding were often unclear. The ISL demonstrated potent anticancer effects by: (1) Inducing apoptosis and autophagy via mechanistic target of rapamycin (mTOR) inhibition and disruption of arachidonic acid pathways; (2) modulating microRNAs (miRs; e.g., miR-374a, miR-200c) to suppress epithelial-mesenchymal transition (EMT); (3) altering hormone receptor [HR; ERα, breast cancer type 1 susceptibility protein (BRCA1)] expression and inhibiting phosphoinositide 3-kinase (PI3K)/protein kinase B (Akt)/mTOR signaling; and (4) reducing angiogenesis [vascular endothelial growth factor (VEGF)/hypoxia-inducible factor-1 alpha (HIF-1α) suppression] and inflammation [cyclooxygenase-2 (COX-2)/nuclear factor-kappa B (NF-κB) inhibition]. Nanoparticle delivery systems (e.g., iRGD-targeted nanoparticles) enhanced ISL’s tumor targeting and efficacy while maintaining low toxicity.

**Conclusions:**

Preclinical evidence highlights ISL’s potential as a multi-target agent against breast cancer progression and metastasis. However, clinical trials are urgently needed to validate its efficacy, safety, and optimal delivery strategies in patients. Future research should prioritize translational studies and combinatorial therapies to bridge the gap between bench and bedside.

## 1. Context

Breast cancer is the most frequently diagnosed malignancy in women and the primary cause of cancer-related mortality among women worldwide. The World Health Organization reports over 2.3 million new cases annually, predominantly in high-income countries, with peak incidence recorded in 2020 (1). It is estimated that breast cancer caused 685,000 deaths in women in 2020, accounting for 16% — or roughly one in six — of all cancer-related deaths in females (2). While the 5-year survival rate for locally invasive breast cancer exceeds 99%, this figure drops sharply to approximately 30% when the disease spreads to distant metastatic sites (3). The survival rate diminishes further if the central nervous system is involved in the metastatic process, with a 1-year survival rate of only 20%, accompanied by a reduced quality of life (4).

Breast cancer is categorized into distinct subtypes according to hormone receptor (HR) and HER2 status. This subtyping is crucial for guiding treatment decisions and predicting disease prognosis. The HR-positive cancers, characterized by the expression of progesterone receptor (PR) and/or estrogen receptor (ER), may be responsive to hormone therapy. Conversely, HER2-positive cancers, which exhibit HER2 overexpression, can be targeted with specific HER2-directed therapies. Common classifications arising from this system include luminal A (ER+/PR+/HER2-), luminal B (ER+/PR+/HER2-/high Ki67), HER2-positive, and triple-negative breast cancer (TNBC), the latter defined by the absence of HER2, PR, and ER expression (5).

The potential of phytoconstituents, naturally occurring compounds derived from medicinal plants, to combat cancer has attracted considerable interest in recent years (2, 6). This interest has stimulated research into medicinal plants as sources of innovative therapeutic options, especially those demonstrating multi-target activity through interactions among multiple components. This approach is well-established in traditional herbal medicine for both disease prevention and treatment (7).

Licorice, derived from the dried roots of *Glycyrrhiza* species, exemplifies such a plant. With a long history of use in both Western and Eastern medical systems for treating diverse ailments, licorice contains various bioactive compounds, including liquiritin, glycyrrhetic acid, glycyrrhizin, and isoliquiritigenin (ISL), a chalcone known to be present in different *Glycyrrhiza* species and used as a folk treatment for conditions like gastric ulcers and coughs (8, 9). The ISL, chemically defined as 2',4',4-trihydroxychalcone, is a prominent chalcone compound naturally occurring within various species of the *Glycyrrhiza* genus (10). The ISL demonstrates a diverse array of biological activities, encompassing antioxidant, antimicrobial, anti-inflammatory, antiviral, hepatoprotective, and anticancer effects (2, 11-13).

Notably, the utilization of ISL within the field of cancer research has gained increasing prominence, particularly concerning its role in the modulation of breast cancer progression (14). Preclinical studies indicate that ISL and its derivatives can inhibit proliferation, migration, invasion, and tumorigenesis in TNBC cells, highlighting its emerging potential as a therapeutic agent against this aggressive subtype of the disease (15-17).

## 2. Objectives

This systematic review aims to comprehensively evaluate the current scientific evidence regarding the multifaceted role of ISL in breast cancer, focusing on its potential for prevention and its ability to modulate key metastatic processes.

## 3. Evidence Acquisition

### 3.1. Search Strategy

This systematic review followed the Cochrane Handbook of Systematic Reviews and PRISMA guidelines (18, 19). The study aimed to assess the effects of ISL on the prevention and management of breast cancer metastases based on preclinical evidence. In March 2025, two investigators (YX and MW) initiated a comprehensive search using MEDLINE/PubMed, Scopus, and Embase for studies published up to 10 May 2023, with a subsequent update on 21 May 2025. Additionally, grey literature was explored through the first 20 pages of Google Scholar, sorted by relevance; the complete search strategy, including MeSH terms and keywords, is provided in Appendix 1. No restrictions regarding geographical location, study design, or language were applied; non-English articles were translated via “Google Translate” (https://translate.google.com/). To further ensure exhaustive coverage, reference lists from eligible studies and related reviews were also examined. References were managed and duplicates removed using Endnote X9 (Thomson Reuters, Philadelphia, USA). Finally, the titles, abstracts, and full texts of the remaining articles were independently screened by investigators to identify studies that met the inclusion criteria.

### 3.2. Selection Criteria

Eligible studies comprised peer-reviewed preclinical investigations (including in vitro, in vivo, and in situ designs) that assessed the impact of ISL treatment on animal models, breast cancer cells, or patient-derived breast cancer tissues using standard laboratory techniques such as the MTT assay, RT-PCR, ELISA, and tumor volume measurement (calculated as 0.5 × length × width^2^). There were no restrictions on the publication period. Studies were excluded if they focused on cancers other than breast cancer or if they involved compounds derived from ISL instead of ISL itself. Furthermore, non-original research formats — such as reviews, letters, personal opinions, communications, book chapters, case reports, and patents — were omitted. Research was also excluded if the full text was not available, if ISL analogs were employed, or if the study did not meet the predefined quality criteria.

### 3.3. Extraction and Quality Evaluation of Data

A data extraction form, created using Microsoft Excel (Microsoft Corporation, Redmond, USA), aided researchers in gathering relevant information. Any disagreements were resolved through discussion. The collected information included: (1) Study details — the author's surname, year of publication, the country where the research was conducted, and the study type (in vitro, in vivo, or in situ); (2) details about the subjects — specifically, the cell lines, animals, and tissues used; (3) information on the intervention — treatment type, dosage, method of administration, delivery system (nanostructure platform), or the specific compound used; and (4) the outcomes assessed: Primary outcomes (positive, negative, or unclear), the mechanisms of action involved, related signaling pathways, and any observed organ or cellular toxicities.

In this study, a high degree of heterogeneity precluded quantitative analysis. Therefore, we have presented our findings in a qualitative synthesis. Given the absence of a universally accepted quality assessment instrument for in vitro studies, a modified version of the Grading of Recommendations Assessment, Development, and Evaluation (GRADE) tool (20) — tailored for in vitro designs — was employed to evaluate study quality, addressing the scarcity of dedicated methodologies. In vitro investigations were then categorized into "high", "moderate", or "low" quality tiers based on a detailed analysis of each study.

To assess the quality of studies utilizing animal models, SYRCLE's Risk of Bias (RoB) tool (21) was implemented. Several potential biases were investigated, including selection, performance, detection, attrition, reporting, and other forms of bias. Furthermore, the Animal Research: Reporting of in vivo Experiments (ARRIVE) Essential 10 checklist (22) was utilized to evaluate the quality of in vivo studies. Ten criteria were assessed for each study individually: The suitability of the study design, sample size, inclusion and exclusion protocols, randomization procedures, blinding application, outcome measurement methodologies, statistical analysis techniques, the characteristics of experimental animals, details of experimental procedures, and the presentation of results. Based on this evaluation, studies that fulfilled 7 to 10 criteria were classified as "high quality", those meeting 4 to 6 criteria were designated as "moderate quality", and those satisfying 1 to 3 criteria were labeled as "low quality". Additional figures and tables pertaining to this research can be found in the Appendix 1 in Supplementary File.

## 4. Results and Discussion

### 4.1. Study Characteristics

**Figure 1.**
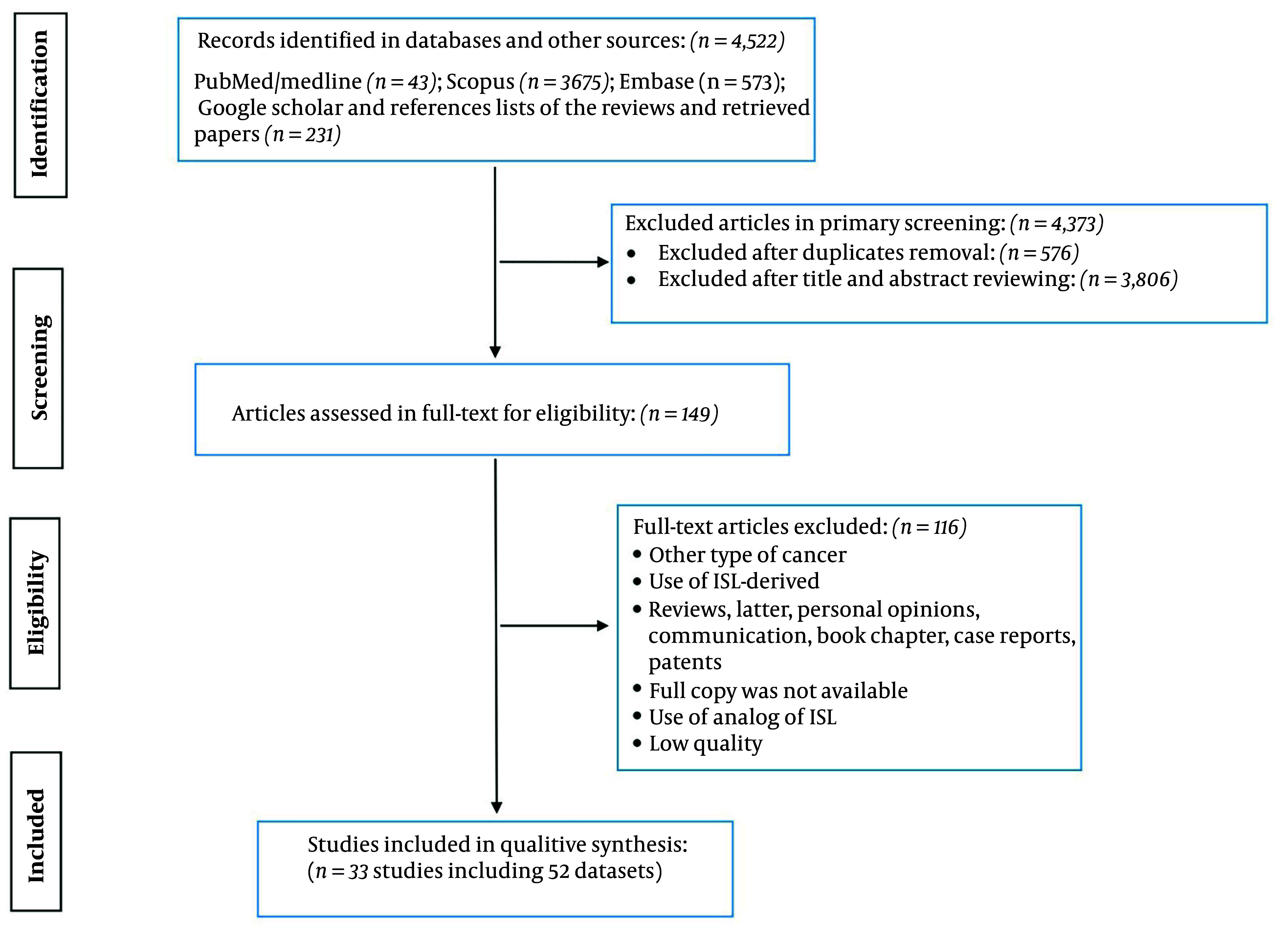
PRISMA flowchart showing the study selection process

A total of 4,522 records were identified across multiple databases, including PubMed/Medline (n = 43), Scopus (n = 3,675), Embase (n = 573), and additional sources such as Google Scholar and reference lists from reviews and retrieved papers to ensure the inclusion of grey literature (n = 231). After primary screening, 4,373 articles were excluded, with 567 removed as duplicates and 3,806 excluded based on title and abstract review. Following full-text assessment for eligibility (n = 149), 117 articles were excluded due to factors such as cancer type mismatch, use of ISL-derived compounds, article type (e.g., reviews, letters, case reports), unavailability of full copies, use of ISL analogs, and low study quality. Ultimately, 33 studies incorporating 52 datasets were included in the qualitative synthesis (Figure 1). 

Table 1 presents an overview of the included studies, outlining their key characteristics, including the authorship, publication year, country, model type, cell line or animal model, treatment details, dosage, administration route, nanostructure platform or compound, mechanism of action, targeted pathway, and toxicity evaluation.

**Table 1.** Summary of the Included Studies ^a, b^

Study	Country	Model	Cell Line or Animal	Treatment; Dose; Route	Nanostructure Platform or Compound	Mechanism	Pathway	Toxicity
**Crone et al. 2019 (** 23 **)**	USA	In vitro	MCF-7 and T-47D cells	ISL ± E2/ICI	N/A	↓ ERα/BRCA1, ±p53, and ↓ proliferation	ERα/BRCA1/p53 expression modulation	No acute toxicity (cell viability preserved)
**Das et al. 2023 (** 24 **)**	India	In vitro	MCF-7 and MDA-MB-231	30 - 40 μM for 24 - 72 h	N/A	↓ Growth and ↑ apoptosis	G2/M arrest, DNA damage, and ↑ apoptosis	-
**Dunlap et al. 2015 (** 25 **)**	USA	In vitro	MCF-10A	1 μM	N/A	↓ P450 1B1 mRNA	Cytokine/TCDD-induced AhR → ↑ P450 1B1	-
**Ganesan et al. 2024 (** 26 **)**	China	In vitro	MDA-MB-231 and others	ISL (40 μM), Blank@ZLH (40 μM), ISL@ZLH NPs (40 μM), and 24 h	ISL@ZLH NPs	↓ Viability, ↓ migration, and ↓ invasion	↓ JAK-STAT (osteoclast inhibition)	-
**Ganesan et al. 2024 (** 27 **)**	China	In vitro	MCF-7 and MDA-MB 231 and others	ISL-NF (0 - 20 μg/mL)	ISL-NFs	↓ Growth, ↓ migration, and ↓ clonogenicity	↓ PI3K/Akt/mTOR, ↑ Casp-3/9, and ↓ MMP-2/9	-
**Ganesan et al. 2024 (** 26 **)**	China	In vivo	Female BALB/c nude mice	ISL (40 μM), Blank@ZLH, ISL@ZLH NPs (20 μM), every 2 d, oral, and 4 wk	ISL@ZLH NPs	↓ Bone metastasis and ↑ survival	↓ PI3K/Akt/mTOR and ↓ MMP-2/9	-
**Ganesan et al. 2024 (** 27 **)**	China	In vivo	Female BALB/c nude mice	ISL (10 mg/kg, qod, and oral); ISL-NF (10 mg/kg, qod, and oral)	ISL-NFs	↓ Tumor growth	Not specified	No significant liver/kidney toxicity
**Gao et al. 2017 (** 28 **)**	Hong Kong	In vitro	MCF-7 and MDA-MB 231 and others	ISL-iRGD NPs/ISL NPs/free ISL/blank NPs (1.6 - 50 μM)	ISL-iRGD NPs	↑ Cytotoxicity, ↑ apoptosis, and iRGD targeting effect	↓ p38, PI3K/Akt, NF-κB, and VEGF/HIF-1α/MMP-2/9	Blank NPs: No cytotoxicity
**Gao et al. 2017 (** 28 **)**	Hong Kong	In vivo	Female nude mice	ISL-iRGD NPs/ISL NPs/free ISL/blank NPs (25 mg/kg)	ISL-iRGD NPs	↓ Tumor growth and ↑ dose efficiency	↓ ERK-1/2 → ↓ CREB → ↓ COX-2	Minimal systemic toxicity; no major organ damage (H&E staining)
**Hsia et al. 2012 (** 29 **)**	Taiwan	In vitro	MDA-MB-231	0.1 - 10 μM	N/A	↓ VEGF, ↓ HIF-1α, ↓ migration, and ↓ MMP-2/9	↓ RANKL/OPG, ↓ COX-2, and ↑ OPG	-
**Lau et al. 2009 (** 30 **)**	Hong Kong	In vitro	MCF-10A cells	1 - 10 μM	N/A	↓ COX-2/PGE2 (PMA-induced)	↓ AA metabolism, ↓ PI3K/Akt, and mitochondrial apoptosis	-
**Lee et al. 2015 (** 31 **)**	Korea	In vitro	MDA-MB-231 and others	0.1, 1, 10, and 20 μM	N/A	↓ RANKL/OPG and ↓ COX-2	↓ AA network (↓ PGE2/20-HETE) and ↑ Casp-3/PARP	-
**Li et al. 2013 (** 15 **)**	China	In vitro	MCF-7 and MDA-MB231	5, 10, and 20 μM	N/A	↓ Proliferation and ↑ apoptosis	↓ NF-κB (p-p65↓), ↑ IκB, and ↓ MAPKs	No significant weight loss or side effects
**Li et al. 2013 (** 15 **)**	China	In vivo	Female athymic BALB/c (nude) mice	50 and 100 mg/kg	N/A	↓ Tumor weight and ↑ apoptosis (TUNEL+)	↓ mTOR → ↑ ULK1 (autophagy/apoptosis)	-
**Li et al. 2022 (** 32 **)**	China	In vitro	others	2.5 - 40 μM	N/A	↓ TNF-α/IL-1β/IL-6 and ↓ iNOS/COX-2	↑ p62 (autophagy) and ↓ VEGF (angiogenesis)	-
**Lin et al. 2020 (** 33 **)**	Taiwan	In vitro	MDA-MB-231	10, 25, and 50 μM	N/A	↑ Apoptosis (cell death)	ERα/β activation → ↑ pS2 mRNA and cytotoxicity at high doses	-
**Lin et al. 2020 (** 33 **)**	Taiwan	In vivo	Female Nude-Foxn1nu mice	ISL (2.5/5 mg/mL, oral, qd, and 2 wk)	N/A	↓ Tumor volume/weight, ↓ Ki-67, and ↑ Casp-3	↓ RECK/MMP9	-
**Maggiolini et al. 2002 (** 34 **)**	Italy	In vitro	MCF7	10 nM	N/A	↑ ERα/β transcription and biphasic proliferation	↓ PIAS3/STAT3/miR-21	-
**Ning et al. 2016 (** 35 **)**	China	In vitro	MDA-MB-231 and others	0 - 40 μM for 24/48 h	N/A	↓ Invasion	Not specified	-
**Ning et al. 2017 (** 36 **)**	China	In vitro	MDA-MB-231 and others	0 - 20 μM for 24 h	N/A	↓ Invasion via ↓ miR-21	miR-374a/PTEN/Akt/β-catenin modulation	-
**Peng et al. 2016 (** 37 **)**	China	In vitro	MCF-7 and MDA-MB-231	Not determined	N/A	↑ Cytotoxicity	↓ Bcl-2, ↑ Bax, ↑ Cyt c, and ↑ Casp-9	-
**Peng et al. 2017 (** 38 **)**	China	In sito	Tissues from 39 breast cancer patients (TMA)	6.25, 12.5, and 25 μM	N/A	↓ Migration and invasion	↑ Bax/Bcl-2 ratio → mitochondrial apoptosis	-
**Peng et al. 2017 (** 38 **)**	China	In vitro	MCF-7 and MDA-MB 231 and others	6.25 - 100 μM	N/A	↓ Proliferation, ↑ apoptosis, and ↓ miR-374a	↑ miR-374a/BAX (apoptosis)	-
**Peng et al. 2020 (** 39 **)**	China	In vitro	MCF-7 and MDA-MB 231 and others	1 - 100 μM	3′,4′,5′,4″-TMC	↑ Apoptosis (TNBC)	↑ miR-200c → ↓ c-Jun	-
**Peng et al. 2020 (** 39 **)**	China	In vivo	Female nude mice	20 and 40 mg/kg/d	3′,4′,5′,4″-TMC	↓ Tumor growth, ↑ BAX, and ↓ miR-374a	Nuclear ISL delivery, ↑ ROS (PDT/TBPI), and targeted cytotoxicity	-
**Peng et al. 2021 (** 40 **)**	Hong Kong	In vitro	MDA-MB-231 and others	-	N/A	↓ EMT and metastasis	GRP78/β-catenin targeting/reversal	-
**Peng et al. 2021 (** 40 **)**	Hong Kong	In vivo	Female nude mice	1mg/mL for 24 h	N/A	↓ Metastasis and tumor growth	↑ HIF-1α degradation → ↓ VEGF/MMP and ↓ VEGFR-2 kinase	-
**Sun et al. 2023 (** 41 **)**	China	In vitro	Others	Intratumoral IT-PEG-RGD (0.1 mg/mL)	ISL NPs, TBPI NPs, and IT-PEG-RGD	↑ Tumor killing (chemo+PDT synergy)	↓ HIF-1α, VEGF/MMP-2/9, and PI3K/Akt/p38/NF-κB	-
**Sun et al. 2023 (** 41 **)**	China	In vivo	Female BALB/cAnU-nu nude mouse	20 - 160 μM	ISL NPs, TBPI NPs, and IT-PEG-RGD	↓ Tumor growth and ↑ drug retention	↓ p-VEGFR-2, ↓ MVD, and ↓ VEGF/MMP-2	-
**Tang et al. 2018 (** 42 **)**	Hong Kong	In vitro	MCF-7 and MDA-MB 231 and others	i.p. 25 mg/kg/d	NISL	↓ Proliferation and ↑ apoptosis	GRP78/β-catenin (CSC targeting)	-
**Tang et al. 2018 (** 42 **)**	Hong Kong	In vivo	Nude mice	5 - 20 μM	NISL	Breast cancer inhibition	↓ miR-25 → ↑ ULK1/autophagy → ↓ ABCG2	Minimal toxicity to normal tissues
**Wang et al. 2013 (** 43 **)**	China	In vitro	MCF-7 and MDA-MB-231	5 - 50 μM	N/A	↓ Angiogenesis (VEGFR-2 blocking)	↓ β-cat./ABCG2/GRP78 + ↑ necrosis (Epi combo)	-
**Wang et al. 2013 (** 44 **)**	China	In vitro	MCF-7 and MDA-MB 231 and others	i.p. 25/50 mg/kg/d	N/A	↓ Motility/invasion (↓ MMPs/VEGF)	↓ miR-25 → ↑ LC3-II/ULK1/BECN1 → ↓ ABCG2	-
**Wang et al. 2013 (** 43 **)**	China	In vivo	Female nude mouse	ISL 25 μM + epirubicin/5FU/taxol (comb.)	N/A	↓ Neo-angiogenesis and ↓ tumor growth (VEGFR-2 blocking)	↑ WIF1 → ↓ Wnt/β-catenin, G0/G1 arrest (CSC suppression)	-
**Wang et al. 2014 (** 45 **)**	Hong Kong	In vitro	MCF-7 and MDA-MB 231 and others	20 - 100 μM	N/A	↑ Chemo-sensitivity (CSC and ↓ GRP78/β-catenin)	ISL-NPs: Enhanced tumor targeting and cytotoxicity	-
**Wang et al. 2014 (** 46 **)**	Hong Kong	In vitro	MCF-7 and others	ISL (50 mg/kg/d) + epirubicin (2.5 mg/kg/wk)	N/A	↑ Autophagy, ↓ miR-25, and ↑ ULK-1 (chemo-sensitization)	ISL-NPs: ↑ Oral uptake → ↑ plasma/tumor ISL levels	-
**Wang et al. 2014 (** 45 **)**	Hong Kong	In vivo	Female NOD/SCID mice	i.p. 2.5 mg/kg/wk + 50 mg/kg/d	N/A	↑ CSC sensitivity (↓ GRP78/β-catenin)	↓ AhR/XRE binding → ↓ CYP1	No apparent toxicity (heart, liver, kidney; confirmed by H&E)
**Wang et al. 2014 (** 46 **)**	Hong Kong	In vivo	Female NOD/SCID mice	25 and 50 μM	N/A	↑ Autophagy, ↓ ABCG2, and ↓ tumor growth	↓ circNAV3 → ↓ brain metastasis risk and ↑ survival	-
**Wang et al. 2015 (** 47 **)**	Hong Kong	In vitro	MCF-7 and MDA-MB 231	50 mg/kg/d × 12 wk	N/A	↓ CSC self-renewal (↑ WIF1 and G0/G1 arrest)	↑ circNAV3 → ↑ brain metastasis and ↓ ISL efficacy	-
**Wang et al. 2015 (** 47 **)**	Hong Kong	In vivo	Female mice	ISL (0 - 80 μM, free/NPs) and blank NPs	N/A	↓ Mammary hyperplasia, cancer, and metastasis	↓ PI3K-Akt-mTOR, ↓ MMP2/9	-
**Wang et al. 2023 (** 48 **)**	Hong Kong	In vitro	MDA-MB-231 and others	Free ISL 40 mg/kg/ISL@ZLH NPs 40 mg/kg	ISL@ZLH NPs	ISL-NPs: ↓ Proliferation/clonogenicity and TNBC-selective	↓ MEK/ERK/C/EBP → ↓ aromatase	-
**Wang et al. 2023 (** 48 **)**	Hong Kong	In vivo	Female BALB/c nude mice	0.1, 1, and 10 μM	ISL@ZLH NPs	ISL-NPs: ↑ Oral bioavailability, ↑ tumor accumulation, and ↑ efficacy	↑ miR-200 c → ↓ PD-L1 mRNA, ↓ ZEB1/2, and ↓ ERK/Src signaling	-
**Wong et al. 2014 (** 49 **)**	Hong Kong	In vitro	MCF-7	10 - 80 μM	N/A	↓ CYP1 via AhR/XRE inhibition	↓ COX-2/CYP4A → ↓ PI3K/Akt → ↑ Casp-3/9 → ↓ MMPs	-
**Xie et al. 2025 (** 17 **)**	China	In vitro	MDA-MB-231 and others	i.p. 50 mg/kg daily, day 3+ post-injection	N/A	↓ circNAV3 → ↓ Brain metastasis and ↑ Survival	↓ PGE2/20-HETE → ↓ PI3K/Akt → ↓ MMP-2/9	-
**Xie et al. 2025 (** 17 **)**	China	In vivo	BALB/c nude female mice	-	N/A	↑ circNAV3 → ↑ brain metastasis (ISL ↓ effect)	ERα/BRCA1/p53 expression modulation	-
**Xu et al. 2025 (** 50 **)**	Hong Kong	In vitro	MCF-7 and MDA-MB 231 and others	Oral	ISL@ZLH NPs	Anti-proliferative and anti-migratory (TNBC cells)	G2/M arrest, DNA damage, and ↑ apoptosis	-
**Xu et al. 2025 (** 50 **)**	Hong Kong	In vivo	Female mice	0.625 - 10 μM	ISL@ZLH NPs	ISL-NPs: ↑ Organ retention, and ↓ proliferation/migration	Cytokine/TCDD-induced AhR → ↑ P450 1B1	-
**Ye et al. 2009 (** 51 **)**	Hong Kong	In vitro	MCF-7	ISL + PTX (comb.)	N/A	↓ Aromatase via MEK/ERK/C/EBP and ↓ proliferation	↓ JAK-STAT (osteoclast inhibition)	-
**Yuan et al. 2024 (** 52 **)**	China	In vitro	MCF-7 and MDA-MB-231	ISL + PTX (comb.)	N/A	↑ CD8+ T-cells, ↓ PD-L1, ↑ miR-200c, and ↑ combo efficacy (with PTX)	↓ PI3K/Akt/mTOR, ↑ Casp-3/9, and ↓ MMP-2/9	-
**Yuan et al. 2024 (** 52 **)**	China	In vivo	Female mice	10, 20, 40 μM	N/A	↓ Tumor, ↓ PD-L1, and ↑ miR-200c (PTX combo)	↓ PI3K/Akt/mTOR and ↓ MMP-2/9	-
**Zheng et al. 2014 (** 16 **)**	China	In vitro	MDA-MB-231 and others	ISL 10/20 mg/kg, oral, 5 × /wk, post-injection	N/A	↑ Anoikis and ↓ metastasis (↓ COX-2/CYP4A and ↑ Casp)	Not specified	-
**Zheng et al. 2014 (** 16 **)**	China	In vivo	Female Balb/cnu/nu mice	ISL ± E2/ICI	N/A	↓ Lung metastasis (↓ PGE2/20-HETE, ↓ PI3K/Akt, and ↓ MMP-2/9)	↓ p38, PI3K/Akt, NF-κB, and VEGF/HIF-1α/MMP-2/9	No acute toxicity (cell viability preserved)

Abbreviations: ISL, isoliquiritigenin; BRCA1, breast cancer type 1 susceptibility protein; AhR, aryl hydrocarbon receptor; NPs, nanoparticles; JAK-STAT, janus kinase-signal transducer and activator of transcription pathway; PI3K, phosphoinositide 3-kinase; Akt, protein kinase B; mTOR, mechanistic target of rapamycin; Casp, cysteine-aspartic proteases; MMP, matrix metalloproteinase; qod, every other day; NF-κB, nuclear factor-kappa B; VEGF, vascular endothelial growth factor; HIF-1α, hypoxia-inducible factor-1 alpha; COX-2, cyclooxygenase-2; H&E, hematoxylin and eosin (staining); RANKL, receptor activator of nuclear factor kappa-Β ligand; OPG, osteoprotegerin; AA, arachidonic acid; PARP, poly (ADP-ribose) polymerase; IκB, inhibitor of kappa B; MAPK, mitogen-activated protein kinase; TUNEL, terminal deoxynucleotidyl transferase dUTP nick end labeling; ERα/β, estrogen receptor alpha/beta; qd, once daily; miR, microRNA; TMC, tetrameth oxychalcone; TNBC, triple-negative breast cancer; ROS, reactive oxygen species; EMT, epithelial-mesenchymal transition; VEGFR-2, vascular endothelial growth factor receptor 2; i.p., intraperitoneal; CSC, cancer stem cell; GRP78, 78 kDa glucose-regulated protein; PTX, paclitaxel.

^a^ MCF-7, MDA-MB-231, etc.: Human breast cancer cell lines.

^b^ ↑: Increase/upregulation; ↓: Decrease/downregulation; →: Leads to/results in.

A total of 52 datasets were included, predominantly in vitro (63.5%) and in vivo (34.5%) studies, with only one in situ analysis. Over half of the studies were published after 2010, indicating increasing interest in this field, particularly between 2010 and 2019 (52%) and 2020 to 2025 (42%). The majority of the datasets originated from China (48%) and Hong Kong (37%), while contributions from other countries were minimal. Among the in vitro studies, nearly half were conducted in China or Hong Kong, with MCF-7 and MDA-MB-231 being the most frequently utilized cell lines, either alone or in combination. Further details on study types, publication years, geographic distribution, and cell line models are provided in Table 2. 

**Table 2.** Characteristics of the Included Studies

Variables	No. (%)
**All datasets**	52 (100)
**Study type**	
In vitro	33 (63.5)
In vivo	18 (34.5)
In situ	1 (2)
Total	52 (100)
**Publication year**	
2000 - 2009	3 (6)
2010 - 2019	27 (52)
2020 - 2025	22 (42)
Total	52 (100)
**Country**	
China	25 (48)
Hong Kong	19 (37)
Taiwan	3 (6)
USA	2 (4)
India	1 (2)
Italy	1 (2)
Korea	1 (2)
Total	52 (100)
**In vitro studies**	33 (100)
Publication year	
2000 - 2009	3 (9)
2010 - 2019	18 (55)
2020 - 2025	12 (36)
Total	33 (100)
Country	
China	15 (45)
Hong Kong	11 (33)
Taiwan	2 (6)
USA	2 (6)
India	1 (3)
Italy	1 (3)
Korea	1 (3)
Total	33 (100)
**Cell line group**	
MCF-7 and MDA-MB-231	6 (18)
MCF-7 and others	2 (6)
MDA-MB-231 and others	8 (24)
MCF-7, MDA-MB-231 and others	8 (24)
MCF-10A	2 (6)
MCF-7 only	3 (9)
MDA-MB-231 only	2 (6)
Other	2 (6)
Total	33 (100)

### 4.2. Risk of Bias and Quality Assessment

Assessment of animal model studies using SYRCLE’s RoB tool revealed a generally low RoB in baseline characteristics, allocation concealment, selective outcome reporting, and other bias sources, indicating strong methodological rigor in these domains. However, sequence generation and random housing were more variable, and blinding procedures were consistently unclear (Appendices 2 and 3 in supplementary File).

The reporting quality of in vitro studies was evaluated using the GRADE framework. Twenty-six of the 33 studies were judged to be of high quality. All studies were positively rated for inconsistency, indirectness, imprecision, publication bias, effect magnitude, and dose effect. However, in the "study limitations" category, seven studies (23, 30-32, 35, 37, 42) displayed incomplete data reporting for some outcomes, leading to a moderate overall quality rating (Appendix 4 in supplementary File).

All in vivo studies met the "High" quality standards of the ARRIVE Essential 10 criteria, but reporting of inclusion/exclusion criteria, randomization, and blinding consistently lacked detail, highlighting areas for improvement despite the robust overall quality (Appendix 5 in supplementary File).

### 4.3. Modulation of Apoptosis and Autophagy via Mechanistic Target of Rapamycin Inhibition

The ISL mediates its antitumor activities in breast cancer cells through multiple interconnected molecular mechanisms. It prominently triggers apoptosis by modulating several key regulators — such as increasing pro-apoptotic proteins (Bax, Bak, Bim), activating cysteine-aspartic proteases (Casp; Casp-3 and Casp-9), and upregulating factors like poly (ADP-ribose) polymerase (PARP) and p53, while downregulating anti-apoptotic signals including Bcl-2, Bcl-xL, survivin, and nuclear factor-kappa B (NF-κB). Simultaneously, ISL impairs angiogenesis by suppressing critical pathways and mediators such as NF-κB, signal transducer and activator of transcription 3 (STAT-3), hypoxia-inducible factor-1 alpha (HIF-1α), vascular endothelial growth factor (VEGF), and vascular endothelial growth factor receptor 2 (VEGFR-2).

**Figure 2.**
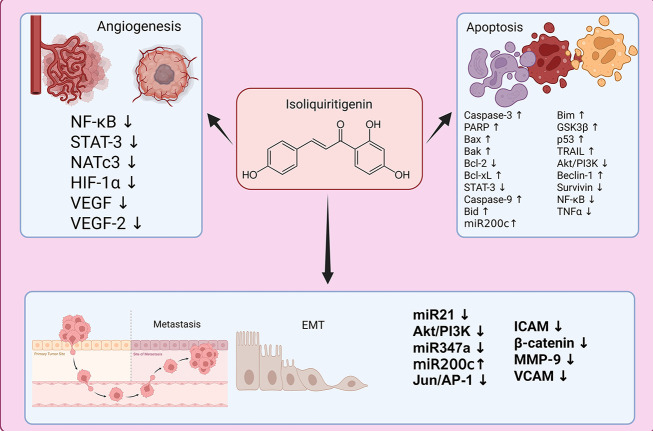
Schematic illustration of the major antitumor mechanisms of isoliquiritigenin (ISL) in breast cancer: The ISL acts on multiple cellular targets to suppress tumor progression by inducing apoptosis (via upregulation of pro-apoptotic factors and inhibition of anti-apoptotic proteins), inhibiting angiogenesis [through downregulation of nuclear factor-kappa B (NF-κB), signal transducer and activator of transcription 3 (STAT-3), hypoxia-inducible factor-1 alpha (HIF-1α), vascular endothelial growth factor (VEGF), and related factors], and preventing metastasis [by reducing the expression of key mediators such as microRNA (miR)-21, phosphoinositide 3-kinase (PI3K)/protein kinase B (Akt), β-catenin, matrix metalloproteinase (MMP)-9, and vascular cell adhesion molecule (VCAM)]. The figure highlights the interrelated molecular pathways affected by ISL, ultimately leading to decreased tumor cell survival, reduced angiogenic potential, and diminished metastatic capability [NATc3, nuclear activating transcription factor 3; VEGFR-2, vascular endothelial growth factor receptor 2; PARP, poly (ADP-ribose) polymerase; TRAIL, TNF-related apoptosis-inducing ligand; GSK3β, glycogen synthase kinase 3 beta; ICAM, intercellular adhesion molecule].

Regarding metastasis, ISL inhibits the invasive and metastatic capabilities of breast cancer cells by downregulating pivotal molecules and pathways — such as miR-21, phosphoinositide 3-kinase (PI3K)/protein kinase B (Akt), miR-347a, Jun/AP-1, intercellular adhesion molecule (ICAM), β-catenin, matrix metalloproteinase (MMP)-9, and vascular cell adhesion molecule (VCAM) (43, 53) — as depicted in Figure 2. Collectively, these actions disrupt cancer cell survival, angiogenic potential, and metastatic progression.

The PI3K/Akt /mechanistic target of rapamycin (mTOR) axis governs a wide array of vital cellular functions — including metabolism, growth, proliferation, programmed cell death, and angiogenesis (54). Signal initiation occurs when extracellular ligands (e.g., insulin or IGFs) engage receptor tyrosine kinases (RTKs) or G-protein-coupled receptors, triggering PI3K activation. The PI3K then phosphorylates phosphatidylinositol-4,5-bisphosphate (PIP_2_) at the inositol 3-position to generate phosphatidylinositol-3,4,5-trisphosphate (PIP_3_). The PIP_3_ recruits Akt and phosphoinositide-dependent kinase-1 (PDK1) to the plasma membrane via their pleckstrin homology domains. There, mTORC2 phosphorylates Akt on Ser 473, inducing a conformational shift that enables PDK1 to phosphorylate Thr 308. Fully activated Akt subsequently phosphorylates substrates at the membrane before relocating to the cytosol and nucleus to promote survival, growth, and proliferation (55).

The lipid phosphatase PTEN counterbalances PI3K by dephosphorylating PIP_3_ back to PIP_2_, thereby blocking Akt membrane recruitment and activation by mTORC2/PDK1 — an action that underlies its tumor-suppressive function (56, 57). In many cancers, PTEN inactivation (by mutation or deletion) leads to PIP_3_ accumulation and persistent Akt signaling (58, 59), with downstream effects even on glucose homeostasis (60).

In breast cancer, aberrations in this pathway are widespread: PIK3CA (p110α), PIK3CB (p110β), and PIK3R1 (p85α) alterations — most notably PIK3CA hotspot mutations E542K, E545K, and H1047R — occur in 30 - 40% of cases, driving constitutive PI3K activity. Concurrent loss of PTEN (5 - 10% of tumors), Akt1 mutations, and RTK amplifications (especially HER2 overexpression in 15 - 20% of tumors) further hyperactivate the cascade, fostering oncogenesis, therapeutic resistance, and poor prognosis, thereby highlighting PI3K/Akt/mTOR as a prime therapeutic target (55).

The Akt’s pro-survival influence is exerted by: (1) Phosphorylating transcription factors (e.g., FOXO family), which suppresses pro-apoptotic gene expression and enhances survival genes such as NF-κB; and (2) phosphorylating key apoptotic regulators — BAD at Ser 136 and Bax at Ser 84 — to inhibit their death-promoting activities and prevent mitochondrial release of cytochrome c and apoptosis-inducing factor (61).

The NF-κB is a family of inducible transcription factors best known for controlling genes central to immune and inflammatory responses (62). In addition to these roles, NF-κB confers resistance to apoptosis triggered by TNF-α, ionizing radiation, or chemotherapeutic agents such as daunorubicin (63), effectively determining whether a cell undergoes programmed death. The essential nature of this anti-apoptotic function was first revealed by Beg and Baltimore, when RelA (p65)-deficient mice succumbed during embryogenesis due to widespread hepatocyte apoptosis (64).

Within the immune system, constitutive NF-κB activity is likewise critical: It drives B-cell differentiation and maintenance, supports thymocyte development, and underpins antigen-specific responses in mature B and T lymphocytes (65). The NF-κB’s inhibition of apoptosis is principally achieved through a tailored transcriptional program that upregulates multiple survival factors. These include the inhibitor of apoptosis proteins cIAP1 and cIAP2, XIAP, TRAF1 and TRAF2, c-FLIP, and anti-apoptotic Bcl-2 family members such as Bcl-XL and A1/Bfl-1, all of which act to block death signaling. Yet, induction of these genes alone does not fully explain NF-κB’s cytoprotective efficacy. Over the past decades, extensive research has revealed that NF-κB also attenuates c-Jun N-terminal kinase (JNK) activation — both downstream of TNFR1 and in response to other apoptotic stimuli — thereby reinforcing its role as a pivotal survival factor (66).

**Figure 3.**
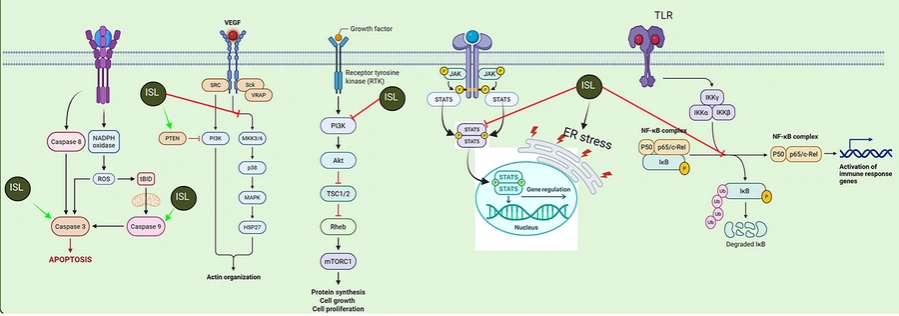
Isoliquiritigenin (ISL) promotes apoptosis through reactive oxygen species (ROS) and cysteine-aspartic proteases (Casp) activation, inhibits cytoskeletal remodeling via vascular endothelial growth factor (VEGF)/phosphoinositide 3-kinase (PI3K)-protein kinase B (Akt), suppresses mechanistic target of rapamycin complex 1 (mTORC1)-driven cell growth, modulates signal transducer and activator of transcription (STAT) 5-dependent gene expression, and blocks toll-like receptor (TLR)/nuclear factor-kappa B (NF-κB)-mediated inflammatory signaling. Red and green arrows indicate inhibitory and stimulatory effects, respectively (MAPK, mitogen-activated protein kinase; IKKα/β/γ, inhibitor of kappa B kinase alpha/beta/gamma; PSD, postsynaptic density; ER stress, endoplasmic reticulum stress; RTK, receptor tyrosine kinase; TSC1/2, tuberous sclerosis complex 1 and 2; NADPH, nicotinamide adenine dinucleotide phosphate (reduced form)).

Our systematic review highlights how ISL leverages complementary mechanisms to induce breast cancer cell death. Li et al. (2013) (15) showed that ISL disrupts the arachidonic acid metabolic network — marked by decreased production of pro-tumorigenic eicosanoids such as PGE_2_ and 20-HETE — thereby attenuating Akt/PI3K signaling upstream of mTOR (Figure 3). In parallel, Lin et al. (2020) (33) demonstrated that ISL markedly reduces mTOR phosphorylation, which both unleashes the intrinsic apoptotic cascade (evidenced by enhanced Casp-3 activation and PARP cleavage) and stimulates ULK1-mediated autophagy, as indicated by p62 accumulation. By concurrently suppressing mTOR’s pro-survival output and overactivating the autophagic machinery, ISL shifts the cellular balance toward catabolic self-digestion and programmed death (Figure 3). This dual assault — impairing anabolic lipid signaling while provoking excessive autophagy — effectively undermines cancer cell proliferation and survival.

### 4.4. MicroRNA Regulation and Epithelial-Mesenchymal Transition Suppression

Over the past several decades, extensive research has established that microRNAs (miRs) — short, non-coding RNA molecules — act as principal post-transcriptional regulators of gene expression by either facilitating the degradation of target messenger RNAs or hindering their translation. Through these mechanisms, miRs orchestrate pivotal biological events such as cellular proliferation, migration, invasion, differentiation, and angiogenesis, all of which are implicated in the onset and progression of breast cancer (67).

High-throughput analytical studies have consistently found that miR-374a is greatly upregulated in numerous malignancies, including head and neck cancer, follicular lymphoma, and small cell lung cancer. In esophageal carcinoma, excessive miR-374a expression is linked to enhanced cell proliferation, achieved through the direct suppression of Axin2, an apoptosis-promoting gene (68). Within the context of breast cancer, particularly in metastatic and TNBC, miR-374a expression is substantially elevated. This upregulation functions via direct inhibition of WIF1, Wnt5a, and notably PTEN — a tumor suppressor that counteracts oncogenic PI3K/Akt signaling pathways. Importantly, higher miR-374a levels have been associated with improved disease-free survival and inversely correlated with invasive tumor characteristics. Moreover, elevated miR-374a has emerged as a potential prognostic marker of favorable outcomes in TNBC populations. Nevertheless, the definitive impact of miR-374a on breast cancer cell proliferation remains ambiguous (69).

The research by Peng et al. (2017, 2020, 2021) (38-40) has consistently highlighted the role of miRs modulation in mediating ISL’s therapeutic effects. Changes in the expression levels of miR-374a and miR-200c play a pivotal role, where upregulation of pro-apoptotic proteins and suppression of epithelial-mesenchymal transition (EMT) are observed (Figure 2). Suppression of EMT directly correlates with reduced metastatic potential, limiting the ability of breast cancer cells to invade surrounding tissues and form secondary tumors (70).

Importantly, across these studies, ISL has shown a remarkably favorable toxicity profile. Cellular viability assays and in vivo histological analyses indicate that ISL is well tolerated, with minimal systemic toxicity or damage to vital organs even at therapeutically effective doses (15, 23, 27, 28, 45, 48).

### 4.5. Disruption of Hormone Receptor Signaling and Cell Cycle Arrest

In addition to its effects on programmed cell death and miRs modulation, ISL also exerts significant influence on signaling pathways and cell growth regulatory mechanisms. Crone et al. (2019) (23) reported that ISL decreases the expression of HRs such as ERα and BRCA1 and exerts variable effects on p53 expression. These alterations are associated with reduced cell proliferation, suggesting that ISL interferes with hormone-dependent growth signals.

Complementing this, Das et al. (2023) (24) found that ISL induces a robust growth inhibitory response in both triple-negative and luminal-A breast cancer cell lines. The compound achieves this by triggering G2/M cell cycle arrest, causing DNA damage, and ultimately leading to apoptosis (70). Such effects highlight the dual role of ISL in both halting cell cycle progression and activating cell death pathways.

### 4.6. Interference with Cellular Metabolism and Inflammatory Pathways

Beyond direct cytotoxic effects, ISL exerts a multifaceted influence on cellular metabolism and inflammatory pathways, significantly broadening its therapeutic potential in cancer treatment. Dunlap et al. (2015) (25) demonstrated that ISL effectively inhibits the mRNA expression of cytochrome P450 1B1 (CYP1B1), a key enzyme involved in the metabolic activation of various procarcinogens, and modulates its induction by inflammatory cytokines in MCF-10A cells. Notably, ISL's mild activation of the aryl hydrocarbon receptor (AhR) pathway suggests that it may competitively interfere with the metabolic activation of procarcinogens within the tumor microenvironment (TME), potentially lowering the overall mutagenic risk and reducing the formation of carcinogenic metabolites (25).

Furthermore, recent investigations by Ganesan et al. (2024) (27) have provided compelling evidence elucidating ISL's suppressive effects on the PI3K/Akt/mTOR signaling cascade, a pivotal pathway critically involved in regulating cell survival, cell cycle progression, metabolism, migration, and metastasis. The observed upregulation of key apoptotic markers, such as Casp-3 and Casp-9, in conjunction with the concurrent downregulation of MMP-2/9, which play a critical role in extracellular matrix degradation and tumor cell invasion, strongly correlates with diminished cancer cell migration and invasion capabilities (Figure 3). 

Importantly, in vivo studies have shown that these molecular events translate into significant therapeutic benefits, including reduced osteolytic bone lesions and extended survival in animal models, all while exhibiting minimal detectable toxicity to vital organs such as the liver and kidneys (26, 27). This favorable toxicity profile, coupled with its diverse mechanisms of action, positions ISL as a promising candidate for further development as a cancer therapeutic.

### 4.7. Nanoparticle-Mediated Enhancement of Isoliquiritigenin Delivery

Drug resistance, a critical impediment to effective cancer therapy, can be either inherent or developed over time, arising from intricate mechanisms that enable neoplastic cells to circumvent the cytotoxic impact of chemotherapeutic drugs (71). Prominent resistance mechanisms encompass enhanced drug efflux mediated by the overexpression of efflux transporters, structural modifications in drug targets, upregulation of DNA repair mechanisms, circumvention of apoptosis, and metabolic adaptations (72). The TME also exerts a significant influence, providing a protective milieu that fosters cancer cell survival and contributes to the development of resistance (73).

Further complicating matters is the inherent heterogeneity of tumors, wherein diverse cellular subpopulations exhibit varying responses to treatment (74). This intratumoral diversity promotes the selective propagation of resistant clones under therapeutic selective pressure, ultimately resulting in treatment failure and disease progression.

Nanoparticle-based drug delivery systems represent a promising strategy to surmount these challenges by improving the stability and bioavailability of therapeutic agents (75). Nanoparticles can be meticulously designed to selectively deliver drugs to the TME and senescent immune cells, thereby minimizing systemic toxicity and augmenting therapeutic outcomes. Attributes such as controlled drug release, protection against enzymatic degradation, and surface functionalization with targeting moieties enable precise drug delivery and sustained therapeutic action (76).

Furthermore, nanoparticles can traverse biological barriers and preferentially accumulate within tumors via the enhanced permeability and retention (EPR) effect, further elevating drug concentrations at the target site (76). This targeted methodology enhances the anticancer activity of drugs and facilitates modulation of immunosenescence within the TME, potentially reversing mechanisms of drug resistance.

The enhancement of ISL delivery and its subsequent therapeutic efficacy has emerged as a prominent area of investigation, driven by the compound's inherent limitations in bioavailability and targeted action. Researchers have explored diverse strategies to overcome these challenges, with a notable emphasis on nanotechnology-based delivery systems (26-28, 41, 48, 50).

Gao et al. (2017) (28) exemplified this approach by utilizing iRGD-targeted nanoparticles to selectively deliver ISL to aggressive breast cancer cells. The iRGD peptide, known for its ability to bind to αvβ3 and αvβ5 integrins overexpressed on tumor cells and endothelial cells within the tumor microenvironment, facilitated enhanced targeting and internalization of the nanoparticles. This sophisticated nanotechnology strategy not only significantly improved cytotoxicity and apoptosis induction in the targeted cancer cells but also enabled effective tumor reduction at considerably lower ISL dosages compared to conventional administration methods. Furthermore, the targeted delivery afforded by the nanoparticles notably spared normal tissues from significant off-target effects and associated toxicities (28).

This innovative approach underscores the profound benefits of nanotechnology in optimizing drug delivery paradigms, enabling precise targeting, controlled release, and protection of the therapeutic payload while simultaneously minimizing detrimental off-target effects on healthy tissues (77, 78). Such targeted delivery mechanisms are crucial for maximizing the Therapeutic Index of ISL and translating its promising in vitro and in vivo anti-cancer potential into clinically relevant outcomes. Further research in this area promises to unlock the full therapeutic potential of ISL and similar compounds.

### 4.8. Anti-angiogenic Effects Through Vascular Endothelial Growth Factor and Hypoxia-inducible Factor-1 alpha Downregulation

Angiogenesis, the development of new blood vessels from pre-existing vasculature (79), represents a tightly orchestrated biological process vital for both physiological homeostasis and the pathogenesis of diverse disease states (80, 81). Precise modulation of angiogenesis hinges upon a complex interplay between pro- and anti-angiogenic signaling molecules (82).

Principal pro-angiogenic mediators encompass VEGF, fibroblast growth factor (FGF), platelet-derived growth factor (PDGF), angiopoietins (Angs), hepatocyte growth factor (HGF), transforming growth factor-β (TGF-β), and MMPs, with the VEGF ligand family serving as the dominant regulator of vascular proliferation (82). This family, comprised of VEGF-A, VEGF-B, VEGF-C, VEGF-D, and placental growth factor, exerts its influence through interaction with endothelial VEGF receptors (VEGFR-1, VEGFR-2, VEGFR-3), transmembrane proteins belonging to the RTK superfamily (83).

Conversely, a cohort of anti-angiogenic factors, including thrombospondin-1, angiostatin, endostatin, vasostatin, tumstatin, interferon-γ, glycosaminoglycans, anti-tissue factor/anti-factor VIIa, and tissue inhibitors of MMPs, serve to counterbalance these pro-angiogenic stimuli (82). Perturbation of this delicate equilibrium precipitates pathological angiogenesis, a hallmark of numerous malignancies (84).

The seminal recognition of angiogenesis as a critical facilitator of tumorigenesis is attributed to Judah Folkman, who posited that neovascularization constitutes an indispensable requirement for the sustained growth of solid neoplasms (85). Malignant cells exploit this process by secreting pro-angiogenic signaling factors, which stimulate the sprouting of new vessels from the adjacent host vasculature, thereby ensuring an adequate supply of oxygen and nutrients to support sustained proliferation and distal dissemination (86).

While tumor-associated endothelial cells (TECs) exhibit unique molecular signatures, a subset of these markers is also observed on endothelial cells within non-neoplastic tissues (87). In the context of breast carcinoma, VEGF emerges as the predominant angiogenic effector (88, 89), frequently exhibiting overexpression prior to the onset of invasive behavior (90) and correlating with diminished clinical outcomes (91). Furthermore, elevated concentrations of serum VEGF are indicative of advanced-stage disease (89, 92), with diminished overall survival observed in patients exhibiting elevated VEGF expression, irrespective of nodal involvement (89).

Additional mediators implicated in breast cancer angiogenesis include VEGFR-2, VEGFR-3, VEGF-D, and VEGF-C, with VEGF-D exhibiting a demonstrated association with lymph node metastasis (93, 94). Beyond VEGF, several pro-angiogenic growth factors, including TGF-β1, pleiotrophin, acidic and basic FGF, placental growth factor, and PDGF, are expressed by invasive breast cancers (95). Furthermore, heightened microvessel density is associated with invasive disease, a greater propensity for metastasis, and diminished patient survival times (91).

In addition to these traditional angiogenic pathways, non-angiogenic mechanisms of vascularization, including vasculogenesis, vascular mimicry, and vessel co-option, also contribute to tumor perfusion. Vasculogenesis, driven by stromal cell-derived factor-1 (SDF1/CXCL12) in response to hypoxia and hypoxia-inducible factor-1 (HIF-1), recruits endothelial progenitor cells or bone marrow-derived hematopoietic cells to facilitate de novo vessel formation (84, 96). Vascular mimicry and vessel co-option, conversely, have been linked to adverse prognoses and enhanced metastatic competence (97, 98). Cumulatively, angiogenic and non-angiogenic vascularization pathways operate in concert within the breast cancer microenvironment, thereby sustaining tumor growth and promoting dissemination.

Hsia et al. (2012) (29) demonstrated that ISL diminishes VEGF secretion and HIF-1α levels by concurrently inhibiting key signaling pathways, including p38 mitogen-activated protein kinase (MAPK), PI3K/Akt, and NF-κB (29). The VEGF, a crucial mediator of angiogenesis — the process by which new blood vessels form from existing vasculature to supply tumors with essential nutrients and oxygen — plays a vital role in tumor growth and metastasis (99, 100). Under hypoxic conditions common within tumors, HIF-1α, a transcription factor, becomes stabilized, leading to increased VEGF expression and further promoting angiogenesis (101). By suppressing both VEGF and HIF-1α, ISL effectively hampers the tumor’s ability to develop new blood vessels.

Moreover, the inhibition of p38 MAPK, PI3K/Akt, and NF-κB — pathways that are integral to cell survival, proliferation, inflammation, and angiogenesis — further amplifies ISL’s anti-angiogenic and anti-inflammatory effects (Figure 3) (29, 33, 43, 44). This multi-targeted approach not only impairs tumor vascularization but also reduces the inflammatory microenvironment that often supports tumor progression and resistance to therapy, highlighting ISL’s potential as a dual-action anticancer agent capable of disrupting both vascular and inflammatory support systems, thereby stifling tumor growth and decreasing metastatic potential.

### 4.9. Anti-inflammatory Activity and Bone Metastasis Control via Cyclooxygenase-2 and Osteoblast Modulation

Investigations by Lau et al. (2009) (30) and Lee et al. (2015) (31) confirmed that ISL modulates cyclooxygenase-2 (COX-2) expression and osteoblast functions, offering potential benefits in controlling both inflammation and bone metastasis in breast cancer. The COX-2 plays a central role in the inflammatory response by catalyzing the production of pro-inflammatory prostaglandins; its overexpression is linked to various cancers, including breast cancer, where it promotes tumor growth, angiogenesis, and metastasis, particularly to bone (102). By downregulating COX-2 expression, ISL mitigates inflammatory signaling pathways that facilitate tumor progression and create a microenvironment conducive to metastasis (30, 31).

Moreover, ISL’s modulation of osteoblast functions suggests that it may influence critical bone remodeling processes, as osteoblasts are responsible for forming new bone tissue — a process that can be hijacked during metastatic spread, resulting in either osteolytic or osteoblastic lesions (103). This regulatory effect on osteoblast activity indicates ISL’s potential to interfere with the establishment and progression of bone metastases, a common and serious complication in advanced breast cancer (17, 26, 27, 40).

Altogether, these findings support the concept that ISL’s anti-inflammatory properties, combined with its ability to influence bone cell function, offer a multifaceted strategy for managing both primary tumor growth and secondary bone metastases, thereby improving patient outcomes and quality of life.

### 4.10. Conclusions

This systematic review underscores the diverse functions of ISL in breast cancer, evidencing its capacity to trigger apoptosis and autophagy via mTOR pathway suppression and arachidonic acid modulation. It also modulates miRs, like miR-374a and miR-200c, to amplify pro-apoptotic signals and inhibit EMT. Furthermore, ISL disrupts HR signaling and interferes with both pro-inflammatory pathways and angiogenesis. Exhibiting a strong toxicity profile across both cellular and in vivo models, ISL presents itself as a compelling prospect for further anticancer therapeutic development, especially if combined with sophisticated delivery techniques and synergistic chemotherapeutic regimens to boost clinical effectiveness and enhance patient outcomes.

### 4.11. Strengths

This study has several strengths, including its comprehensive synthesis of preclinical research on ISL in breast cancer. The review cohesively presents ISL's diverse anti-cancer mechanisms — encompassing the induction of apoptosis and autophagy, modulation of miR expression, and disruption of HR and inflammatory signaling pathways — drawing upon evidence from both in vitro and in vivo studies. Furthermore, the work emphasizes innovative delivery systems, such as nanoparticle formulations, which augment ISL's therapeutic efficacy, establishing a robust framework to guide subsequent clinical investigations.

### 4.12. Limitations

This study also has some limitations. A key challenge within the existing research landscape is the scarcity of clinical trial data confirming ISL's effectiveness and safety in patients with breast cancer. While preclinical investigations have extensively documented ISL's radical scavenging, antimicrobial, anti-inflammatory, and antitumor properties, its translation into clinical applications remains uncertain. Moreover, despite ISL demonstrating potential for improving chemosensitivity in diverse breast cancer models, complete evaluations of its toxicity profile — especially concerning long-term safety and potential adverse effects — are currently insufficient, hindering its immediate therapeutic application.

### 4.13. Future Research

Future research should prioritize translating preclinical successes to the clinic through comprehensive clinical trials that rigorously assess ISL's therapeutic effects on patient outcomes. Furthermore, optimizing ISL delivery methods, such as exploring nanoparticle-based oral formulations to improve tumor targeting and minimize toxicity, warrants significant attention. Investigating synergistic combinations of ISL with standard chemotherapeutic agents may also enhance its anticancer effectiveness, ultimately facilitating the development of more effective and personalized treatment approaches for individuals with breast cancer.

ijpr-24-1-165301.pdf

## Data Availability

The datasets used in the current study are available from the corresponding author upon reasonable request.
